# Pathophysiological Features of Remodeling in Vascular Diseases: Impact of Inhibitor of DNA-Binding/Differentiation-3 and Estrogenic Endocrine Disruptors

**DOI:** 10.3390/medsci13010002

**Published:** 2024-12-26

**Authors:** Vincent Avecilla, Mayur Doke, Sandeep Appunni, Muni Rubens, Venkataraghavan Ramamoorthy, Jayanta Kumar Das

**Affiliations:** 1 Robert Stempel College of Public Health & Social Work, Florida International University, Miami, FL 33199, USA; 2 Avecilla Consulting LLC, Miami, FL 33131, USA; 3 Diabetes Research Institute, University of Miami, Miami, FL 33136, USA; 4 Department of Biochemistry, Government Medical College, Kozhikode 673008, Kerala, India; 5 Baptist Health South Florida, Miami Gardens, FL 33176, USA; 6 Department of Health and Natural Sciences, Florida Memorial University, Miami Gardens, FL 33054, USA

**Keywords:** endocrine disruptors, *ID3*, transcriptional regulator, vascular remodeling

## Abstract

Vascular diseases, such as hypertension, atherosclerosis, cerebrovascular, and peripheral arterial diseases, present major clinical and public health challenges, largely due to their common underlying process: vascular remodeling. This process involves structural alterations in blood vessels, driven by a variety of molecular mechanisms. The inhibitor of DNA-binding/differentiation-3 (*ID3*), a crucial member of ID family of transcriptional regulators, has been identified as a key player in vascular biology, significantly impacting the progression of these diseases. This review explores the role of *ID3* in vascular remodeling, emphasizing its involvement in processes such as apoptosis, cell proliferation, and extracellular matrix regulation. Furthermore, we examine how oxidative stress, intensified by exposure to estrogenic endocrine disruptors (EEDs) like polychlorinated biphenyls (PCBs) and bisphenol A (BPA), affects *ID3* activity and contributes to vascular disease. Understanding the interaction between *ID3* signaling and EED exposure provides critical insights into the molecular mechanisms underlying vascular remodeling and its role in the development and progression of vascular diseases.

## 1. Introduction

Vascular diseases, including hypertension, atherosclerosis, cerebrovascular, and peripheral arterial diseases, are major global health challenges, responsible for about 32% of all deaths worldwide. In 2019 alone, cardiovascular diseases (CVDs) claimed 17.9 million lives, with heart attacks and strokes accounting for 85% of these fatalities [1,2,3]. The burden is particularly high in low- and middle-income countries, where over three-quarters of these deaths occur. Despite advances in prevention and treatment, factors such as population growth, aging, obesity, and diabetes continue to drive the rise in CVD-related deaths, especially in regions like Africa, Asia, Eastern Europe, and South America [4,5,6].

Vascular remodeling is a vital process in cardiovascular health, involving structural changes in blood vessels that affect their function. This remodeling is commonly seen in conditions like hypertension, atherosclerosis, and heart failure, where it can lead to increased vascular resistance and impaired blood flow [7,8]. The process is driven by cellular and molecular mechanisms such as vascular smooth muscle cell proliferation, elastin degradation, and extracellular matrix calcification, with inflammation and oxidative stress playing key roles [7,9]. While vascular remodeling can initially be an adaptive response to maintain blood flow, it often becomes maladaptive, contributing to disease progression and complications like acute coronary events and heart failure [8,10]. Despite significant research, there are still knowledge gaps, particularly in understanding the long-term effects of vascular remodeling and identifying effective therapeutic targets [9,11].

Recent research indicates that estrogenic endocrine disruptors (EEDs) and the inhibitor of DNA-binding/differentiation-3 (*ID3*) protein are pivotal in vascular remodeling and dysfunction. EEDs, synthetic compounds that imitate or obstruct estrogen signaling, are recognized for impairing vascular endothelial cell function by attaching to estrogen receptors, elevating oxidative stress, and modifying essential pathways including vascular endothelial growth factor (VEGF) signaling and cell adhesion [12,13,14,15,16,17,18,19,20]. These disturbances lead to endothelial dysfunction, inflammatory responses, and structural alterations in blood vessels, resulting in chronic vascular inflammation and heightened risk of cardiovascular illnesses [21,22,23,24,25,26,27].

The *ID3*, a transcriptional regulator, has been associated with vascular remodeling. The *ID3* facilitates vascular smooth muscle cell proliferation, extracellular matrix calcification, and endothelial cell activation, which are crucial for vascular homeostasis but may induce pathological alterations in the presence of oxidative stress or exposure to EEDs [20,28,29,30,31]. Significantly, environmental endocrine disruptors such as polychlorinated biphenyl 153 (PCB153) have been demonstrated to enhance *ID3* expression, establishing a connection between environmental exposures and vascular diseases [20,31].

Thus, we tried to decipher the existing understanding of how *ID3* may impact these various components in vascular remodeling that lead to alterations in the vessel causing obstruction and further damage. Since many of these vascular remodeling mechanisms are oxidative stress-dependent [12], exposure to EEDs may also contribute to perturbations that take place during vascular remodeling. Estrogenic endocrine disruptors are mainly human-made chemicals discovered in our environment that act by modifying hormonal events. EEDs such as estrogenic polychlorinated biphenyls (PCBs) [13], bisphenol A (BPA) [14], phthalates [15], and diethylstilbestrol (DES) [16] have been linked to affecting metabolic health throughout vital phases of development and adulthood. Epidemiological studies have narrated links between estrogenic endocrine disruptors and various cardiovascular and vascular diseases [17,18,19]. Based on current discoveries that established *ID3-dependent* endothelial cell activation via estrogenic PCB153 exposure, we will discuss how exposure to EEDs may contribute to complex vascular lesions, in which vascular disease manifests via *ID3* [20]. Previously, we elucidated the association between *ID3* and EEDs in metabolic syndrome (MetS) perturbations via adipose tissue that bioaccumulate, which can modify various chronic diseases such as vascular, neurological, cancer, and autoimmune ones [21,28]. This may help clarify additional factors contributing to vascular alternations in the blood vessel. Additional studies in these capacities may uncover innovative avenues of beneficial modalities as well as deliver prevention, control, and treatment approaches of vascular remodeling with exposure to estrogenic endocrine disruptors and the dysregulation of *ID3*.

This review aims to explore the complex role of *ID3* in vascular remodeling, which is a crucial factor in the development of cardiac diseases. We will investigate the data that connect *ID3* to crucial elements of vascular remodeling, including apoptosis, proliferation, alterations in the extracellular matrix, and cellular migration. In addition, we will investigate the possible effects of exposure to EEDs on *ID3*-mediated pathways and their role in causing changes in blood vessels. Our goal is to gain a new understanding of the molecular mechanisms that cause vascular remodeling and how it relates to the development of vascular diseases by examining the relationship between *ID3* and EEDs. The purpose of this review is to offer a thorough comprehension of the current level of knowledge in this field and emphasize prospective directions for future study and therapeutic intervention.

## 2. Inhibitor of DNA-Binding/Differentiation-3 (*ID3*)

*ID3* belongs to a family of small proteins that includes *ID1*, *ID2*, *ID3*, and *ID4*. This family exhibits significant amino acid sequence homology (69–78%) within their helix–loop–helix (HLH) domain, while the remaining regions are highly divergent. Initially identified as a serum-inducible early-response gene, *ID3* shows a peak in transcriptional activity at 1 h, followed by a biphasic expression pattern with a secondary surge at 24 h during tissue regeneration after injury [32,33]. Experimental studies have highlighted the critical role of *ID3* in cell differentiation and embryonic development. Dual knockout of the Id1 and Id3 in mice results in cardiac abnormalities, impaired neuronal differentiation, and defective brain vascularization, which are embryonically lethal [34,35]. While *ID3* is highly expressed in embryonic tissue, its levels decrease as cells differentiate. Notably, *ID3* expression can be induced by various stimuli across multiple cell types [36]. The *ID3* is a transcriptional regulator recognized for preventing stem cell differentiation and the stimulation of cell cycle progression. A member of ID family of helix–loop–helix proteins programmed by an immediate early gene receptive to oxidative stress and mitogenic signals, *ID3* via accumulative evidence suggests that it may be involved in vascular remodeling, a process in which alterations in the structure of the vessel and vessel wall occur including four processes: cell death, cell growth, synthesis or degradation of the extracellular matrix, and cell migration [12,31,37,38]. Vascular remodeling is supported upon dynamic interactions between hemodynamic stimuli, vasoactive substances, and growth factors, which may contribute to the pathophysiology of cardiac diseases [39]. For instance, *ID3* has previously been studied in combination with lipoxygenase (12/15-LO), which is demonstrated to produce pro-inflammatory alterations in blood vessels that lead to the progression of atherosclerosis [40]. Furthermore, 12/15-LO has been seen as an essential intermediary of vascular smooth muscle cell (VSMC) growth and its growth-promoting effect, which is mediated by *ID3* transcription [41]. Concerning these molecular interactions with blood vessels, it is also significant that *ID3* is important to embryonic vasculogenesis as well as endothelial cell activation [31,37,38].

In contrast to its pro-apoptotic effects in several cancer cell types, *ID3* overexpression in endothelial cells has been demonstrated to enhance cell survival and inhibit cell death. When compared to cells of the wild type, *ID3* dramatically lowers apoptosis in human cerebral microvascular endothelial cells [42]. By upregulating markers like CD133, VEGFR3, and CD34, it also improves stemness [43,44]. Furthermore, when co-cultured with smooth muscle cells, *ID3* overexpression promotes the development of 3-D microvascular defects [42]. By modifying chromatin accessibility to cell death pathways [45], interacting with E-proteins to affect transcriptional activity [46], and controlling the transcription of cell survival genes, *ID3* most likely promotes survival mechanistically.

**Figure 1:**
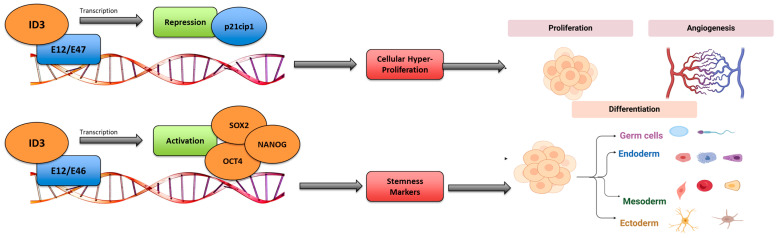
*ID3* transcriptional regulation. Figure illustrates various roles in cellular functions such as apoptosis, angiogenesis, differentiation, and cellular growth. The *ID3* regulates transcription genes such as *p21cip1*, *OCT4*, *SOX2*, and *NANOG*. Adapted from our previously published work (Avecilla, Doke, and Felty, 2017) [21] with permission.

Previously, Felty and Das demonstrated that vascular endothelial cells exposed to E2 (17β-estradiol) or polychlorinated biphenyl 153 (PCB153) exhibited increased protein phosphorylation, endothelial neovascularization, and elevated *ID3* expression [47]. Furthermore, PCB153 increased oxidative stress or ROS (reactive oxygen species) that facilitate *ID3* expression [47]. Estrogenic chemical exposure has been demonstrated to increase ROS, altering neighboring DNA essential for transcriptional stimulation of cell-growing genes. *ID3* protein–protein communications arise through the HLH motif. During this, ID proteins dimerize and block the DNA-binding activity of basic HLH transcription factors such as E-proteins, which include E2-2, E12, E47, and HEB, programmed by the transcription factor 12 (*TCF12*) and transcription factor 3-4 (*TCF3-4*) gene [48]. *ID3* protein–protein communications can control transcription by E-proteins, inhibiting the subsequent binding and stimulation of target gene promoters. Furthermore, E-proteins subdue the expression of embryonic genes SRY-box 2 (*SOX2*), Octamer-Binding Transcription 4 (*OCT4*), and Nanog Homeobox (*NANOG*), leading to cell differentiation as demonstrated in Figure 1 [48]. Additionally, *ID3* stimulates cells to pass via cell cycle borders by hindering the expression of the cell cycle inhibitor gene *p21Cip21*. Research has established that ectopic overexpression of *ID3* enhanced *SOX2* and *OCT4* expression and resulted in a positive cell population for molecular stem cell markers CD133^+^ VEGFR3^+^ CD34^+^. Based on these outcomes, *ID3* maintains cells in a noncommittal or undifferentiated state by blocking the repression of pluripotency influences by *TCF3* [49,50]. Since *ID3* regulates genes associated with stemness and cell proliferation, it is likely that EEDs could drive vascular remodeling by promoting uncontrolled proliferation through *ID3*, thereby contributing to the formation of lesions and the development of various vascular diseases.

## 3. Vascular Remodeling

### 3.1. Vessel Patterning, Inflammation, and Vascular Dysfunction

Building on the role of *ID3* in vascular remodeling and its regulation by environmental and molecular factors, this section focuses on the structural and functional changes in blood vessels that characterize vascular remodeling. Atherosclerotic lesions, often associated with vascular diseases, are defined by disruptions to normal vessel architecture and function. These alterations are influenced by developmental processes, with insights from developmental biology offering a deeper understanding of gene-specific roles in the pathogenesis of vascular diseases. By integrating embryological insights with genetic research, we can better understand how genes such as *ID3* contribute to vascular remodeling and the progression of vascular diseases [50,51,52]. The endothelium plays a crucial role in maintaining vascular health, acting as a non-thrombogenic barrier that supports blood flow while exhibiting anti-inflammatory and growth-inhibitory properties. Dysregulation of endothelial function increases vascular tone, promotes inflammation, and accelerates the progression of conditions like hypertension and atherosclerosis [50,53].

Endothelial dysfunction, marked by the overexpression of adhesion molecules such as VCAM-1, facilitates leukocyte adhesion and migration, thereby worsening vascular inflammation [54]. This dysfunction is driven by oxidized lipids, which activate NF-κB pathways and stimulate the production of pro-inflammatory cytokines like TNF-α and IL-1β [54,55]. Cytokines such as TNF-α are key mediators of systemic inflammation, influencing leukocyte activation, metalloproteinase production, and CAM expression. Chemokines such as MCP-1 further exacerbate inflammation by regulating macrophage infiltration into lesions and promoting inflammatory signaling via NF-κB and AP-1 pathways [56]. These processes collectively drive smooth muscle cell proliferation and extracellular matrix calcification, contributing to arterial stiffening and vascular remodeling. Chronic inflammation also disrupts the balance between pro-inflammatory and anti-inflammatory interleukins, with cytokines like IL-6 being closely linked to increased risks of coronary artery disease and myocardial infarction [57]

### 3.2. Extracellular Matrix Remodeling and RAAS in Vascular Lesions

Arterial stiffness and disruptions in the extracellular matrix (ECM) are critical contributors to vascular remodeling, leading to increased peripheral resistance and adverse cardiovascular outcomes. The ECM provides essential structural and biochemical support to cells, influencing processes such as proliferation, differentiation, and migration [58]. Cells interact with ECM cues to adapt their microenvironment, but this adaptability can become dysregulated in conditions like pulmonary arterial hypertension, atherosclerosis, and peripheral arterial disease. Matrix metalloproteinases (MMPs) play a pivotal role in maintaining ECM homeostasis and mediating cell-to-cell and cell-to-matrix interactions. However, inflammatory responses and oxidative stress can dysregulate these interactions, further driving vascular remodeling [59,60].

The renin–angiotensin–aldosterone system (RAAS) also plays a crucial role in vascular remodeling by amplifying inflammation and oxidative stress, thereby exacerbating cardiovascular damage. Components such as aldosterone and angiotensin II (Ang-II) are central to maintaining cardiovascular homeostasis but can also contribute to disease pathogenesis. For instance, decreased expression of Angiotensin II Type 2 Receptor (AT2R) alongside increased expression of Angiotensin II Type 1 Receptor (AT1R) is linked to endothelial dysfunction, vascular hypertrophy, and arterial stiffness [61,62,63]. The intricate interplay between the ECM, MMPs, and RAAS highlights the complexity of vascular remodeling [61,64]. A thorough understanding of these mechanisms is essential for developing precise therapeutic strategies to slow disease progression and improve patient outcomes.

## 4. *ID3* and Vascular Diseases

Involvement of *ID3* in vascular diseases has been studied through various types of models. Previously, Forrest et al. demonstrated during vascular lesion development in rats an alternate isoform of the *ID3*. Early expression of *ID3* in lesion formation when the proliferation indicator of the neo-intima is highest and stimulates smooth muscle cell (SMC) proliferation alongside S-phase entry inhibits transcription of the cell cycle inhibitor p21Cip1. Overall, this delivers a novel indication that regulated intron preservation can modify a pathologic process in vivo [65]. The *ID3* has also been studied with lipoxygenase (12/15-LO), which is recognized to create pro-inflammatory alterations in blood vessels leading to atherosclerosis development [66]. Indeed, 12/15LO in the vessel wall is demonstrated to increase in MetS and diabetes mellitus as demonstrated in animal models. Higher expression of 12/15LO increases the proliferation of cultured vascular smooth muscle cell (VSMC) proliferation, an effect that is intermediated by *ID3*. Deliri et al.’s results demonstrated *p21cip1* as a conceivable target of the 12/15LO-*ID3* pathways and indicate that a variation in this pathway may have beneficial placement for affecting the higher prevalence of restenosis in diabetic mellitus patients [67]. Using ubiquitous E-proteins as a stimulus, Matsumura et al. determined that *ID3* and an *ID3* (*ID3*a) novel isoform were cloned. Balloon injury revealed that *ID3*a was abundantly expressed throughout the neo-intimal layer. These outcomes deliver an indication that *ID3* gene may represent a significant method by which neo-intimal SMC growth is weakened throughout the formation of vascular lesions [68]. VSMCs from principal aortic leukocyte-type 12/15-LO transgenic, leukocyte-type 12/15-LO knockout (KO), and control mice were equally plated and assayed for growth, *ID3* transcription, and *ID3* protein expression. This established that transgenic 12/15-LO VSMCs cultivated rapidly while KO 12/15-LO VSMCs cultivated slower compared to control VSMCs [41].

Reactive oxygen species (ROS) are involved in the irregular growth of numerous cell types. Angiotensin-II (Ang-II) is one of the most applicable inducers of oxidative stress in the vasculature. It was demonstrated that *ID3* overexpression of antisense through transfection in VSMCs entirely obliterated Ang-II- and X/XO-induced cell proliferation. Overexpression of sense *ID3*, Ang-II, and X/XO also demonstrated the downregulated protein expression of p21WAF1/Cip1, p53, and p27Kip1. Overexpression of sense *ID3* and Ang-II caused hyper-phosphorylation of the retinoblastoma protein. Ang-II furthermore induced the proliferation of VSMCs via the production of superoxide, which increases the expression of *ID3*. Downstream mitogenic processing is regulated by *ID3* through p21WAF1/Cip1, p53, and p27Kip1. These overall results reveal a redox-sensitive pathway involved in growth control [69]. The *ID3* also plays a role in high-fat-diet-stimulated visceral adipose VEGFA expression, depot expansion, and microvascular blood volume [57]. The *ID3* is essential to obesity due to its demonstration of stimulating angiogenesis and is considered an important factor of HFD (high-fat-diet)-induced visceral adiposity [70]. Knockout (KO) *ID3* mice demonstrated a significant protective outcome from HFD-induced visceral fat depot development when compared to the control. Furthermore, adipose tissue neighboring main arteries (perivascular adipose tissue or PVAT) demonstrated evidence of specifying vessel insulation and support. Developing evidence elucidates that PVAT mediates artery pathology and physiology, such as the development of atherosclerosis. It was previously demonstrated by Harmon et al. that C57BL6 mice with the B cell-specific insufficient *ID3* had larger IgM production and visceral adipose tissue B-1b cells and less inflammation of adipose tissue when compared to those of WT littermate controls. Comparably, Prasad et al. showed that *ID3* mediates B-1b cells in PVAT demonstrating how *ID3* is involved with various types of adipose tissue [71,72]. Furthermore, patients with heritable PAH and pulmonary arteries in BMPR-II mutant mice exhibited reduced *ID3* levels compared with those of control subjects. Particularly, *ID1* and *ID3* are critical downstream effectors of BMP signaling in PASMCs and regulate the PASMC proliferation through cell cycle inhibition [73]. Maier et al. observed the signaling pathway BMPR-II in hPASMCs with no fundamental cardiovascular disease (non-PAH hPASMCs). Female non-PAH hPASMCs demonstrated reduced messenger RNA and protein expression of BMPR-II; the signaling intermediary *SMAD1*; downstream genes; and inhibitors of DNA-binding proteins, *ID1* and *ID3*. Induction of phospho-Smad1/5/8 and ID proteins by BMP4 was also reduced in female hPASMCs. *BMP4* induced proliferation in female, but not male, hPASMCs. The results demonstrated that the estrogen-dependent suppression of BMPR-II signaling in non-PAH hPASMCs from women provides a proliferative phenotype in hPASMCs that may affect women with PAH [74].

Coronary artery disease, which is a disease or injury to the heart’s main blood vessels, causes approximately one out of seven deaths in the United States. Population-based studies have discovered single-nucleotide polymorphism (SNP) rs11574 in the coding region of *ID3* gene is linked with atherosclerosis in the Diabetes Heart Study [75]. *ID3* SNP rs11574 has also demonstrated a significant link to coronary artery disease for Caucasians besides a condensed range of African Americans and Hispanics [76]. Previously, genes that have been demonstrated to be involved in CAD include *PLA2G7*, *LFNG*, *PADI4*, *ARG1*, *FOLR3*, *NFIL3 IL1R2*, *MGAM*, and *ID3*, which were differentially expressed [77]. These genes showed a connection to CAD via statistical and biological pathway analysis. Shi et al. determined that pathways linked to immune responses, particularly neutrophil degranulation, were correlated with coronary heart disease [77]. Outgrowth endothelial cells (OECs), an endothelial progenitor cell (EPC) subtype able to figure vessel structures, have been introduced as a novel therapeutic model for cell-driven treatments for stroke. OECs demonstrated greater expressions *of CCL2*, *ID3*, *IGF-1*, *MMP9*, *TGFBR1*, *TNFAIP2*, *TNF*, and *TGFB1*. The results demonstrated that OECs from stroke patients demonstrate greater levels of factors (pro-angiogenic) at early stages, declining in developed OECs when they convert more similarly to established microvascular endothelial cells [78]. Furthermore, *ID3* may be a predictive factor for stroke, which is sudden death to brain cells due to inadequate blood flow. Expression levels of genes (*STK3*, *ANTXR2*, *PDK4*, *CD163*, *MAL*, *ID3*, *PLXDC2*, *GRAP*, *CTSZ*, *KIF1B*) were data-mined, matched between groups, and assessed for their predicative capability at each time reference in 23 ischemic stroke patients. The results demonstrated that candidate gene expression levels were able to distinguish between stroke patients and controls with points of understanding and specificity above 90% across all three time frames. Based on these lines of evidence, these results suggest the analytic power of the design of differential expression in an independent population of patients and additionally indicate that it is temporally constant over the first 24 h of stroke [79]. *ID3* expression also demonstrated a molecular stemness marker comprising CD133^+^ VEGFR3^+^CD34^+^ cells. Positive protein expression of *ID3*, CD34, and VEGFR3 and increased expression of pluripotent transcription factors *SOX2* and *OCT4* were demonstrated due to SU5416 exposure. Overexpressing *ID3* cells reinforces the development of a 3-D microvascular lesion co-cultured with smooth muscle cells. Further investigations into how stem-like cells apply *ID3* may lead to novel possibilities for improved comprehension of the mechanisms [49]. Overall, the impact of *ID3* on cardiac illnesses has been thoroughly investigated using different models, uncovering its pivotal role in vascular remodeling, endothelial dysfunction, and regulation of adipose tissue. The *ID3* has a crucial role in the development of vascular diseases, starting with the establishment of arterial lesions and extending to its involvement in pro-inflammatory pathways in atherosclerosis. In addition, the way *ID3* interacts with metabolic syndrome, oxidative stress, and immunological responses demonstrates the complex influence it has on cardiovascular health. The results emphasize the potential of *ID3* as a target for therapy and a biomarker for early identification and intervention in vascular diseases. This opens new possibilities for developing innovative approaches to prevent and treat these conditions.

## 5. EEDs in Vascular Disease

There is a rising trepidation that estrogenic endocrine disruptors may contribute to the pathogenesis of vascular disease such as estrogenic polychlorinated biphenyls (PCBs) and bisphenol A (BPA). Previous population-based studies have shown a linkage between EED exposure and higher vascular disease risk. Studies have revealed an association between PCBs and hypertensive participants in the NHANES (National Health and Nutrition Examination Survey) (1999–2004) [80]. Demographic characteristics demonstrated greater concentrations among those with hypertension. Further analyses identified categories of PCBs significantly linked with increased risk of hypertension (PCB 66, 101, 118, 28, and 187) [80]. Additionally, levels of environmental chemicals were associated with hypertension in a population sample of men and women. When these environmental chemicals were treated as constant variables and regulated for only gender, two PCBs (PCB 105 and 118) were linked to prevalent hypertension. These results further strengthened the experimental findings that these pollutants might influence blood pressure [81]. Additionally, a Spanish cohort of university graduates encompassed 14,521 participants, originally hypertension-free, which was followed up for a median of 8.3 years. Concentration levels of polychlorinated biphenyls taken and calculated in food samples eaten in Spain were used to calculate nutritional consumption. Following adjustment for overall energy intake and possible confounders, members in the fifth quintile of total PCB consumption were at higher risk of hypertension development when compared with members in the first quintile. The results demonstrated that nutritional consumption of PCBs assessed using a food rate questionnaire showed a greater risk association with hypertension development during follow-up [82].

Endothelial cells, which line blood vessels and play a critical role in vascular function, are highly susceptible to the effects of endocrine-disrupting chemicals (EDCs) [83,84]. These cells express various hormone receptors, making them direct targets for EDCs, such as those that bind to estrogen receptors and disrupt normal signaling pathways [83,84]. This disruption can lead to altered cell adhesion, particularly through the vitronectin receptor (integrin αvβ3), compromising vascular integrity and function [85]. Additionally, EDCs can interfere with vascular endothelial growth factor (VEGF) secretion, disrupting angiogenesis, which is vital for maintaining vascular health [22,83]. By affecting endothelial cell proliferation, migration, and differentiation, EDCs contribute to vascular remodeling, leading to structural and functional changes in blood vessels [22]. Furthermore, many EDCs induce oxidative stress in endothelial cells, resulting in cellular damage and dysfunction, and promote inflammatory responses that contribute to chronic vascular inflammation, a precursor to atherosclerosis and other cardiovascular diseases [23]. Exposure to EDCs, such as bisphenol A (BPA), can also lead to endothelial dysfunction, an early and crucial event in cardiovascular disease development, and disrupt the transcription of genes essential for vascular health [24,25]. Recent research reports suggest that endocrine disorders, influenced by EDC exposure, are increasingly recognized as significant contributors to pathological changes linked to endothelial dysfunction, further highlighting the critical role of these chemicals in cardiovascular disease progression [21,26,86].

The effects of PCB126 on vascular inflammation have been linked to hepatic dysfunction utilizing a liver injury mouse model. Mice were exposed to PCB126 (0.5 mg/kg) and analyzed for inflammatory, calorimetric, and metabolic parameters. MCD (methionine–choline-deficient)-diet-fed mice demonstrated steatosis, suggestive of a compromised liver. PCB126 induced steatosis irrespective of the diet type, but only the MCD+PCB126 group exhibited steatohepatitis and fibrosis. Furthermore, PCB126 exposure in MCD-diet-fed mice led to increased plasma inflammatory markers such as pro-atherogenic trimethylamine-N-oxide (TMAO), PAI-1, and ICAM-1, suggesting inflammation of the peripheral vasculature that is characteristic of atherosclerosis. Taken together, data provide new evidence of a link between a compromised liver, PCB-mediated hepatic inflammation, and vascular inflammatory markers, suggesting that environmental pollutants can promote crosstalk between different organ systems, leading to inflammatory disease pathologies [87]. Modifications of epigenetic indicators can be stimulated by environmental pollutant exposure and may contribute to the possibility of vascular disease. Exposure to coplanar PCBs 77 and 126 stimulated H3K9 trimethyl demethylase jumonji domain-containing protein 2B (JMJD2B) expression and nuclear factor-kappa B (NF-κB) subunit p65, and it triggered NF-κB signaling, as demonstrated by the nuclear translocation of p65. Based on these lines of evidence, it is suggested that coplanar PCBs may utilize the toxicity of endothelial cells via changes in histone alterations [88].

PCB153 has been exhibited to bind to estrogen receptor alpha, increase the development of ROS, and prompt vessel development in endothelial cells. Because PCB153-induced phenotypic changes are analogous to those of estradiol, it is hypothesized that PCB153 stimulates redox-signaling pathways common to 17β-estradiol [89]. Previously, it was determined that planar and coplanar PCBs induce the expression of various genes compared to estradiol. The results demonstrated that vascular endothelial cell exposure to levels of estrogenic PCBs stimulates gene networks involved in the process of inflammation and adhesion. This suggests that PCBs can stimulate the formation of vascular lesions by triggering gene networks involved in endothelial cell growth, adhesion, and pro-inflammatory molecules differently from natural estrogens [90].

It also has been shown that a linkage between exposure to BPA, another EED, and many diseases that affect the vascular system exists. BPA is a chemical used commonly in food and drink packaging. Shankar et al. assessed the association between urinary BPA levels and PAD in a nationally representative sample of U.S. adults. A positive association was demonstrated between growing concentrations of urinary BPA and PAD before and after adjusting for confounders [91]. Recruitment from a population-based sample of adolescents and young adults based on a mass urine screening was used to define the association between serum levels of BPA and carotid intima-media thickness (CIMT). After confounding factors were controlled, results showed a 1-unit increase in natural log BPA was significantly associated with an increase in mean CIMT. Greater serum levels of BPA were linked with higher CIMT in this study of adolescents and young adults [92].

Various animal models have verified links between BPA and the vasculature. BPA-treated rabbits exhibited insulin resistance, hepatic steatosis, and noticeable adipose accumulation. Exposure to BPA also triggered myocardial damage and augmented atherosclerotic development in the aortic arch with advanced lesion areas (69%) and macrophage number (86%) [93]. Furthermore, BPA has demonstrated induced proliferation and collagen production in a concentration-dependent manner, as revealed by wound healing assay, MTT, and collagen assay. Additional study results demonstrated that BPA acts as a promoting factor in the proliferative process and collagen production of cardiac fibroblasts via activating ERK1/2 [94]. Changes in the rates of ventricular contraction indicate higher parasympathetic activity detected in males. Lower systolic blood pressure was observed in males exposed to BPA and in females from the highest BPA exposure group. Based on these lines of evidence, the results show noteworthy changes (sex-specific) in gene expression that were consistent with the observed BPA exposure-related phenotypic alterations in the extracellular matrix, altered autonomic responses, lipid metabolism, and cardiac remodeling [95].

Urinary BPA concentrations can also be linked to arterial hypertension. Eight-week-old mice were administered with BPA via drinking water. Mice established high blood pressure and an increase in arterial angiotensin II (Ang-II); significant eNOS-dependent superoxide and peroxynitrite accumulation impairment of acetylcholine; an 8.7-fold increase in eNOS mRNA and protein; and Ang-II inhibition with reduced oxidative stress, normalized blood pressure, and endothelium-dependent relaxation. These lines of evidence suggest that Ang-II separates eNOS and contributes to BPA-induced endothelial dysfunction by fostering nitrosative and oxidative stress [96]. Lind and Lind examined levels of phthalate metabolites and BPA in a study related to atherosclerosis. In the population-based study, the prevalence of overt plaques and echogenicity of carotid artery plaques were recorded by ultrasound in both carotid arteries. BPA and phthalate metabolites were analyzed in serum. Monomethyl phthalate was associated with carotid plaques in an inverted U-shaped manner. This pattern was important after adjustment for BMI, gender, blood glucose, blood pressure, HDL and LDL cholesterol, serum triglycerides, smoking, antihypertensive treatment, and statin use (*p* = 0.004). Some phthalates and BPA were also related to the echogenicity of the plaques, suggesting a role for plaque-associated chemicals in atherosclerosis [97].

Estrogens are a key regulator of cardiovascular function, primarily through its interaction with estrogen receptors ERα, ERβ, and GPER, which play crucial roles in cellular processes vital for maintaining heart health and mitigating diseases such as idiopathic pulmonary hypertension (IPH) [98]. By binding to these receptors, estrogenic ligands modulate gene expression within cardiovascular tissues, significantly impacting cellular proliferation, inflammation, and vascular remodeling. For instance, estrogens have been shown to delay the onset of cardiac hypertrophy by reducing the phosphorylation of p38-mitogen-activated protein kinase (MAPK) under pressure overload conditions, highlighting its protective effects, particularly in postmenopausal women [99]. Moreover, estrogens enhance the expression of endothelial nitric oxide synthase (eNOS) and inducible nitric oxide synthase (iNOS), promoting nitric oxide (NO) production, which is critical for vasodilation and inflammation control [100,101]. Estrogens’ beneficial effects extend to pulmonary arterial hypertension, where it mitigates the severity of the condition through mechanisms involving ERα, ERβ, and GPER, thereby promoting vasodilation and protecting against vascular remodeling [98,101,102]. The tissue-specific responses to estrogens, especially in pulmonary arteries, are crucial in vascular diseases, affecting vascular tone, endothelial function, and smooth muscle cell proliferation. Estrogens also play a significant role in modulating mitochondrial function by reducing ROS production and stabilizing mitochondrial structures, further supporting cardiovascular health. However, the estrogens paradox in IPH, where estrogens can act both as a protective factor and a risk factor, particularly in women, underscores the complexity of its role in cardiovascular diseases [23,103]. This balance of estrogenic signaling and tissue-specific effects is susceptible to disruption by EEDs, which can mimic or block estrogenic ligands, leading to altered receptor activation and gene expression [13,14,15,16]. Such interference by EECs can diminish the protective effects of estrogens, potentially worsening conditions like IPH and disrupting the regulatory processes essential for cardiovascular health.

In conclusion, the evidence presented in this review highlights the substantial influence of estrogenic endocrine disruptors, including PCBs and BPA, on the restructuring of blood vessels and the development of heart and lung illnesses. Further work is needed to understand the molecular processes by which these contaminants affect important genes and signaling pathways related to vascular health. Gaining insight into the interaction between environmental contaminants and the process of restructuring blood vessels is essential for creating methods to prevent and treat cardiac illnesses. Future research should prioritize the clarification of the specific functions of these endocrine disruptors in vascular disease and investigate new therapies to mitigate their harmful impacts.

## 6. *ID3* and EEDs in Vascular Remodeling

The ID proteins, including *ID1*, *ID2*, and *ID3*, have been extensively studied for their interactions with environmental toxicants such as BPA and PCBs [21]. These proteins play crucial roles in cellular processes like differentiation, proliferation, and development. Research indicates that while exposure to BPA, particularly in utero, does not significantly alter *ID2* mRNA levels in adult testes, exposure to diethylstilbestrol (DES) has been shown to reduce mRNA expression of genes involved in Sertoli cell differentiation, including *ID2* [21]. Similarly, PCB exposure, especially to Aroclor 1254, has been associated with changes in various ID gene expression organs, such as the liver and kidney, where *ID2* mRNA expression increases in a dose-dependent manner, potentially serving as a marker for thyroid-disrupting chemical exposure [104]. The *ID3*, in particular, has drawn attention due to its association with PCB153, which has been linked to increased *ID3* protein expression and oxidative DNA damage in blood vessels. The *ID3* is implicated in the development of microvascular lesions and the survival of vascular endothelial cells in response to environmental chemicals like PCB153 [27,105]. The development of PCB-induced microvascular lesions is driven by a complex interplay of mechanisms, including oxidative stress, *ID3* overexpression, and aberrant neovascularization [106]. PCB153 exposure leads to increased production of reactive oxygen species in vascular endothelial cells, triggering microvascular damage [106]. This oxidative stress further promotes *ID3* overexpression, enhancing endothelial cell survival, neovascularization, and the development of a proliferative endothelial phenotype. The process is driven by ROS-mediated signaling [31], *ID3*-dependent pathways, and Pyk2 activation, all contributing to microvascular lesion formation [107]. Moreover, PCBs disrupt endothelial function by altering gene expression, promoting cell proliferation, and affecting vascular tone regulation, while metabolic disturbances, such as disrupted thyroid hormone homeostasis and altered glucose metabolism, exacerbate microvascular damage [47,108,109]. The estrogenic activity of certain PCBs may also interact with nuclear receptors, leading to hormonal disruptions that contribute to vascular remodeling and dysfunction. In summary, the interaction between ID proteins and environmental toxicants like BPA and PCBs plays a significant role in health, influencing conditions such as metabolic health, neurocognitive disorders, and vascular health, with PCB-induced microvascular lesions resulting from a multifaceted interplay of oxidative stress, *ID3* overexpression, neovascularization, endothelial dysfunction, and metabolic disturbances [47,108,109].

Previously, an estrogen-induced oxidant mechanism regulated differentiation in endothelial cells into tube-like structures via *ID3* was examined. Superoxide scavenger MnSOD and hydrogen peroxide scavenger catalase overexpression inhibited the formation of the tube in estrogen-treated endothelial cells. Due to the non-specificity of tube formation on matrigel for endothelial cells, Porther et al. demonstrated results that more accurately represent in vivo tube formation through co-culture modeling [31]. They observed that estrogens enhanced the phosphorylation of *ID3*, which was attenuated by treatments with *N*-acetylcysteine and catalase. RNA interference determined the role of *ID3* in tube formation and demonstrated *ID3* siRNA to inhibit tube development in cells exposed to estrogens. Overall, tube formation that is a result of estrogens requires *ID3* factors [31]. Enhanced neovascularization has been involved with the growth development or advancement of proliferative vascular lesions. Overall, the results demonstrated that a highly tube-branched PCB-induced ROS-facilitated neovascular phenotype was shown to be dependent on *ID3* and PYK2. PCB153 treatment additionally caused endothelial spheroids to increase in size under conditions generally used for the clonal selection of stem cell spheroids. PCB153 treatment also increased both serine and tyrosine phosphorylation of the endothelial *ID3* [47].

Moreover, EEDs interfere with hormonal signaling pathways, often leading to oxidative stress and inflammation. One of the key mechanisms by which EEDs exert their effects is through the generation of ROS, which disrupts cellular homeostasis and influences gene expression. The *ID3* has been identified as a downstream target of oxidative stress induced by EED exposure. Studies have shown that EEDs such as PCBs and BPA increase ROS production, which activates signaling pathways like NF-κB and AP-1, resulting in the upregulation of *ID3* expression in vascular endothelial cells and smooth muscle cells [44,110]. The interplay between EEDs and *ID3* is particularly pronounced in vascular remodeling processes. PCB153, a frequently examined endocrine disruptor, has been demonstrated to elevate *ID3* expression by augmenting oxidative stress and activating transcriptional regulators. The *ID3* affects endothelial cell function by facilitating cell cycle progression and inhibiting differentiation, resulting in endothelial dysfunction and vascular remodeling [44,110]. EEDs interfere with VEGF signaling and NO production, activities that are further intensified by *ID3* activity, leading to compromised angiogenesis and heightened vascular permeability [111]. These findings underscore a synergistic link between EED-induced oxidative stress and *ID3*-mediated vascular alterations. Comprehending this interplay yields essential insights into the role of environmental exposures in the development of vascular illnesses, including atherosclerosis and pulmonary arterial hypertension.

**Figure 2:**
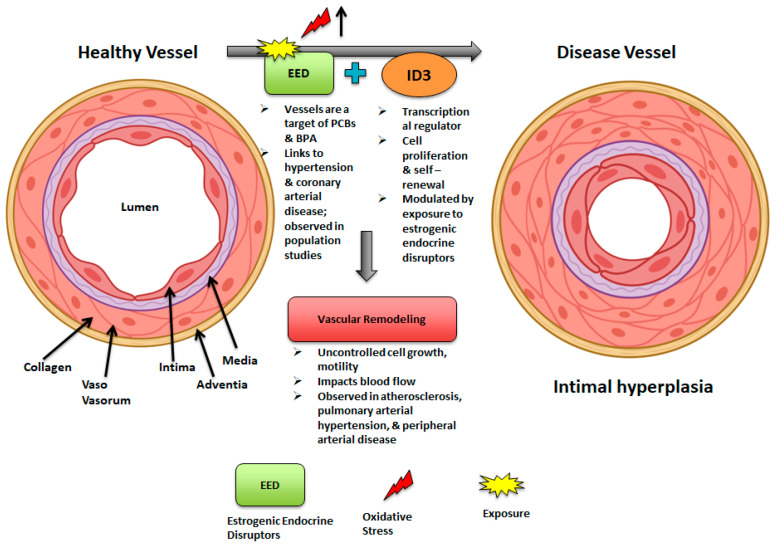
EED and *ID3* influence on vessels. Left panel shows healthy vessel and right panel shows potential effect on diseased vessel with exposure to EEDs and modulation of transcriptional regulator *ID3*. Exposure of EEDs increases oxidative stress causing modulation of *ID3*, which mediates vascular remodeling; uncontrolled cell growth; and narrowed lumen, which impacts blood flow.

**Figure 3:**
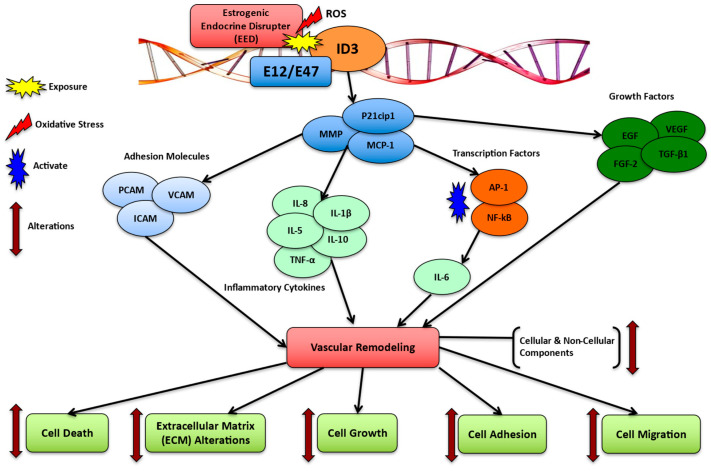
Mechanisms of *ID3* and EEDs in vascular remodeling. Exposure to EEDs increases oxidative stress causing the modulation/activation of the transcriptional regulator, *ID3*. Mediation of various vascular remodeling factors via *ID3* may elucidate alterations in cell death, the extracellular matrix, cell growth, cell adhesion, and cell migration.

The interaction between EED exposure and *ID3* signaling involves a network of pathways driving oxidative stress, inflammation, endothelial dysfunction, and vascular remodeling. EEDs like PCBs elevate ROS levels, triggering oxidative stress that activates *ID3*, a redox-sensitive molecule [112]. This activation facilitates the acquisition of stem cell-like molecular signatures in endothelial cells, which may aggravate vascular remodeling. EED-induced oxidative stress also initiates pro-inflammatory responses, characterized by increased levels of IL-6 and TNF-α. The *ID3* plays a key role in this process by regulating cytokine production, thereby promoting angiogenesis and stem cell-like properties [72,113]. In endothelial dysfunction, *ID3* influences VEGF signaling, boosting endothelial cell proliferation and contributing to microvascular lesion formation. It also promotes a stem cell-like phenotype (CD133^+^ VEGFR3^+^ CD34^+^) and may intensify mitochondrial dysfunction caused by EEDs by disrupting the balance of mitochondrial fission and fusion [72,113]. Additionally, the interplay between EED exposure and *ID3* drives vascular remodeling by facilitating 3-D microvascular lesion formation and modulating BMP signaling to enhance smooth muscle cell proliferation. *ID3*-mediated endothelial stemness further accelerates vascular changes, similarly to the mechanisms observed in pulmonary arterial hypertension. In summary, the interaction between EEDs and *ID3* establishes a self-reinforcing loop of oxidative stress, inflammation, and vascular remodeling, contributing to the progression of vascular diseases as demonstrated in Figure 2. Table 1 and Table 2 offer a detailed overview of the interaction between EEDs and *ID3* in vascular pathology. Table 1 delineates the participation of EEDs, *ID3*, and their synergistic effects in diverse vascular illnesses, whereas Table 2 elucidates the principal molecular pathways, including oxidative stress, inflammation, and endothelial dysfunction, that facilitate vascular remodeling. These tables provide a systematic framework to enhance the comprehension of the evidence and trends presented in this study. Additionally, since microvascular diseases are categorized by unwarranted vessel growth, it is rational that neovascularization that is demonstrated as estrogen-produced contributes to the development of microvascular lesions. A proposition for how *ID3* overexpression in endothelial cells contributes to the progression of an estrogen-induced neovascular phenotype with emphasis on PYK2 kinase was shown. Overexpression of *ID3* increased spheroid growth, cell migration, and neovascularization of human cerebral microvascular endothelial cells, hCMEC/D3. Furthermore, overexpressed *ID3* cells presented important estrogen-induced G2/M phase conversion. These results imply that PYK2 signals *ID3* expression and *ID3* is crucial for neovascularization in hCMEC/D3 cells via estrogen induction [106]. The *ID3* is also involved with both pulmonary arterial hypertension (PAH) and hemorrhagic telangiectasia (HHT). Because PAH can be revealed in patients who have a function loss of the activin A receptor-like type 1 or *ACVRL1* gene, this certain mutation can lead to HHT, an autosomal dominant inherited disease resulting in arteriovenous malformations [114]. As demonstrated in Figure 3, exposure to EEDs may trigger *ID3* to modulate mechanisms in vascular remodeling [84]. These alterations may affect various cellular and non-cellular components such as cell growth, cell adhesion, cell migration, ECM alterations, and cell death as demonstrated in Figure 3. Moreover, Table 1 outlines the role of EEDs, *ID3*, and their combined impact across diverse vascular diseases. It shows the methods via which these factors lead to diseases such as atherosclerosis, pulmonary arterial hypertension, coronary artery disease, and cerebrovascular disease, supported by relevant references.

Table 2 delineates the molecular pathways affected by EEDs and *ID3*, including oxidative stress, inflammation, vascular smooth muscle cell activity, and endothelial dysfunction. It offers a comprehensive examination of the interactions among various pathways that facilitate vascular remodeling and pathology, accompanied by relevant references.

## 7. Conclusions

In this manuscript, we have thoroughly explored the current data on the role of *ID3* in vascular remodeling and its interplay with environmental chemicals, particularly EEDs. Our analysis elucidates the role of *ID3* in mediating critical processes, including vascular smooth muscle cell proliferation, endothelial dysfunction, and extracellular matrix remodeling, which contribute to vascular pathologies such as atherosclerosis, coronary artery disease, cerebrovascular disease, and pulmonary arterial hypertension.

We have examined the pathways by which EEDs affect *ID3* expression and function, elucidating how environmental exposures intensify vascular remodeling and facilitate disease development. The capacity of EEDs to provoke oxidative stress and modify signaling pathways via *ID3* offers essential understanding of the convergence of environmental and biological variables that impair vascular homeostasis.

This analysis highlights the necessity for more research to elucidate the molecular processes connecting *ID3*, EEDs, and vascular remodeling. Disentangling these connections may facilitate the identification of innovative treatment targets and preventive methods for vascular disorders. Future research should concentrate on pinpointing particular mechanisms and therapies to alleviate the effects of *ID3* and EED-induced vascular remodeling, providing viable remedies to combat the increasing burden of these harmful disorders.

## Figures and Tables

**Table 1:** Table categorizing evidence by disease classification.

Vascular Disorder	Role of EEDs	Role of *ID3*	Combined Effects	References
Atherosclerosis	Induces oxidative stress and promotes inflammation	Regulates vascular smooth muscle cell (VSMC) proliferation and inflammation	Synergistic contribution to plaque formation	Kirkley & Sargis, 2014; Wassmann et al., 2010; Yang et al., 2014 [17,44,110]
Pulmonary Arterial Hypertension	Disrupts endothelial signaling and inhibits angiogenesis	Drives vascular remodeling and endothelial damage	May exacerbate vascular lesion formation	Crosswhite & Sun, 2014; Doke, 2018; Endocrine Disrupting Chemicals: Threats to Human Health|IPEN [111,112,113]
Coronary Artery Disease	Modifies vascular tone and triggers inflammatory pathways	Contributes to smooth muscle and endothelial dysfunction	Potentially accelerates lesion development	Doran et al., 2010; Helsley & Zhou, 2017; Manichaikul et al., 2014 [72,73,114]

**Table 2:** Table Organizing Evidence by Mechanisms of Action.

Mechanism	EED Role	*ID3* Role	Combined Role	References
Oxidative Stress	Elevates ROS production and disrupts signaling pathways	Facilitates ROS-induced endothelial dysfunction	Synergistically amplifies vascular oxidative damage	Lința et al., 2024; Valaitienė & Laučytė-Cibulskienė, 2024; Yilmaz et al., 2019 [33,115,116]
Inflammation	Stimulates cytokine production and leukocyte adhesion	Modulates inflammatory mediators and NF-κB activity	Intensifies inflammation, exacerbating vascular remodeling	Hansen et al., 2015; McDonald et al., 1997; Mussbacher et al., 2023 [117,118,119]
Vascular Smooth Muscle Cells	Promotes calcification and uncontrolled proliferation	Induces VSMC phenotype switching	Accelerates arterial stiffness and plaque development	Cao et al., 2022; Durham et al., 2018; Guan et al., 2024 [120,121,122]
Endothelial Dysfunction	Disrupts VEGF signaling and nitric oxide synthesis pathways	Impairs endothelial integrity and angiogenesis	Aggravates vascular remodeling and increases permeability	Avecilla et al., 2024; Migliaccio et al., 2021; Muntean et al., 2024 [81,123,124]
